# Changes in entropy on polarized-sensitive optical coherence tomography images after therapeutic subthreshold micropulse laser for diabetic macular edema: A pilot study

**DOI:** 10.1371/journal.pone.0257000

**Published:** 2021-09-13

**Authors:** Koji Ueda, Tomoyasu Shiraya, Fumiyuki Araki, Yohei Hashimoto, Motoshi Yamamoto, Masahiro Yamanari, Takashi Ueta, Takahiro Minami, Nobuyori Aoki, Satoshi Sugiyama, Han Peng Zhou, Kiyohito Totsuka, Taku Toyama, Koichiro Sugimoto, Ryo Obata, Satoshi Kato

**Affiliations:** 1 Department of Ophthalmology, Graduate School of Medicine, The University of Tokyo, Tokyo, Japan; 2 Department of Clinical Epidemiology and Health Economics, School of Public Health, The University of Tokyo, Bunkyo-ku, Tokyo, Japan; 3 Engineering Department, Tomey Corporation, Nagoya, Aichi, Japan; Roskamp Institute, UNITED STATES

## Abstract

**Purpose:**

To investigate the dynamics of the healing process after therapeutic subthreshold micropulse laser (SMPL) for diabetic macular edema (DME) using polarization-sensitive optical coherence tomography (PS-OCT).

**Methods:**

Patients with treatment-native or previously-treated DME were prospectively imaged using PS-OCT at baseline, 1, 2, 3, and 6 months. The following outcomes were evaluated: changes in the entropy value per unit area (pixel^2^) in the retinal pigment epithelium (RPE) on the B-scan image; changes in the entropy value in each stratified layer (retina, RPE, choroid) based on the ETDRS grid circle overlaid with *en face* entropy mapping, not only the whole ETDRS grid area but also a sector irradiated by the SMPL; and the relationship between edema reduction and entropy changes.

**Results:**

A total of 11 eyes of 11 consecutive DME patients were enrolled. No visible signs of SMPL treatment were detected on PS-OCT images. The entropy value per unit area (pixel^2^) in the RPE tended to decrease at 3 and 6 months from baseline (35.8 ± 17.0 vs 26.1 ± 9.8, P = 0.14; vs 28.2 ± 18.3, P = 0.14). Based on the *en face* entropy mapping, the overall entropy value did not change in each layer in the whole ETDRS grid; however, decrease of entropy in the RPE was observed at 2, 3, and 6 months post-treatment within the SMPL-irradiated sectors (P < 0.01, each). There was a positive correlation between the change rate of retinal thickness and that of entropy in the RPE within the SMPL-irradiated sector at 6 months (r^2^ = 0.19, P = 0.039).

**Conclusion:**

Entropy measured using PS-OCT may be a new parameter that facilitates objective monitoring of SMPL-induced functional changes in the RPE that could not previously be assessed directly. This may contribute to a more promising therapeutic evaluation of DME.

**Clinical trial:**

This clinical study was registered in UMIN-CTR (ID: UMIN000042420).

## Introduction

Diabetic macular edema (DME) is the leading cause of decreased visual acuity in patients with diabetic retinopathy [1]. With regard to the treatment of DME, several prospective and randomized studies have shown that subthreshold micropulse laser (SMPL) is a more effective and minimally invasive therapy for DME compared to the conventional macular laser, that is, the modified Early Treatment Diabetic Retinopathy Study (mETDRS) photocoagulation method [2–7]. In addition, SMPL improves or stabilizes visual acuity and reduces macular thickness without laser scarring and retinal damage as shown by clinical and fundoscopic examinations performed using spectral-domain optical coherence tomography (SD-OCT) and fundus autofluorescence (FAF) imaging [8, 9].

Although the molecular mechanisms of SMPL underlying the treatment success are not yet completely understood, photothermal stimulation to the retinal pigment epithelium (RPE) is believed to activate heat shock proteins (HSP), which are associated with cell function, autoregulation, and immunomodulation of the retina [10, 11]. Intriguingly, an *in vivo* study using mouse models demonstrated that SMPL possibly induces monocyte recruitment to the RPE followed by hematopoietic progenitor cell homing, which may contribute to one of the therapeutic effects [12]. Moreover, SMPL downregulates the expression of vascular endothelial growth factor (VEGF) [13, 14] and inflammatory cytokines that are mainly produced by retinal macrophages, such as macrophage inflammatory proteins-1α [14].

In addition to retinal thickness and macular volume, the following DME-related and choroidal changes in patients with diabetes have been investigated using SD-OCT: reflectivity of intra/subretinal fluid, integrity of the outer retina including the external limiting membrane or ellipsoid zone, presence of intraretinal hyperreflective foci (HRF), and changes in the thickness and structure of the choroid [15–19]. It was previously reported that SMPL may cause some molecular and functional changes in the retina [10, 13, 14]; however, the current SD-OCT technology has limitations regarding elucidation of these mechanisms. In this study, polarization-sensitive optical coherence tomography (PS-OCT) has been employed as an extension to conventional OCT to observe the depth-resolved polarization properties of a sample by measuring the state of a polarized electromagnetic field [20, 21]. As melanin-loaded structures randomly modify the state of polarization, PS-OCT can indicate whether and the extent to which the integrity of these structures is preserved and provide information on their conformation in a detailed manner [22, 23]. Since melanin is found mostly in the RPE cell organelles, analyzing the polarization states detected by PS-OCT together with the information obtained from conventional SD-OCT images, tissue-specific depolarizing properties of the RPE can be determined [24]. In fact, using PS-OCT images, the properties of coagulated lesions on the RPE and its healing responses after macular laser with pattern scanning laser for DME have been reported [25].

Almost all of the previous studies of PS-OCT have used degree of polarization uniformity (DOPU) as a depolarization metric to detect the melanin [22–25]. Although it has been proven that DOPU was useful in a lot of clinical studies, it has recently been known that DOPU depends on the incident state of polarization [26], potentially limiting the capability of DOPU to evaluate the depolarization quantitatively. As one of the approaches to overcome the limitation in DOPU, Yamanari et al. introduced a mathematical framework to compute “entropy” that represents the randomness of the polarization property in an advantageous way, both physically and mathematically [27]. We have previously reported that Entropy in PS-OCT is a parameter that is independent of the incident polarization state and that in principle it is highly reproducible in retake [28]. Furthermore, high reproducibility of entropy values in normal eyes also has been reported [29].

We believe that improving our understanding of the morphological changes occurring after SMPL treatment is of great value, and in this study, we address this issue using a prototype PS-OCT system designed for clinical studies. The aim of this study was to evaluate the dynamics of the healing process and to provide further insights into the polarization-scrambling changes in the (sensory) retina, RPE, and choroid that are induced by SMPL performed for the treatment of DME.

## Materials and methods

### Patients and inclusion

**Fig 1:**
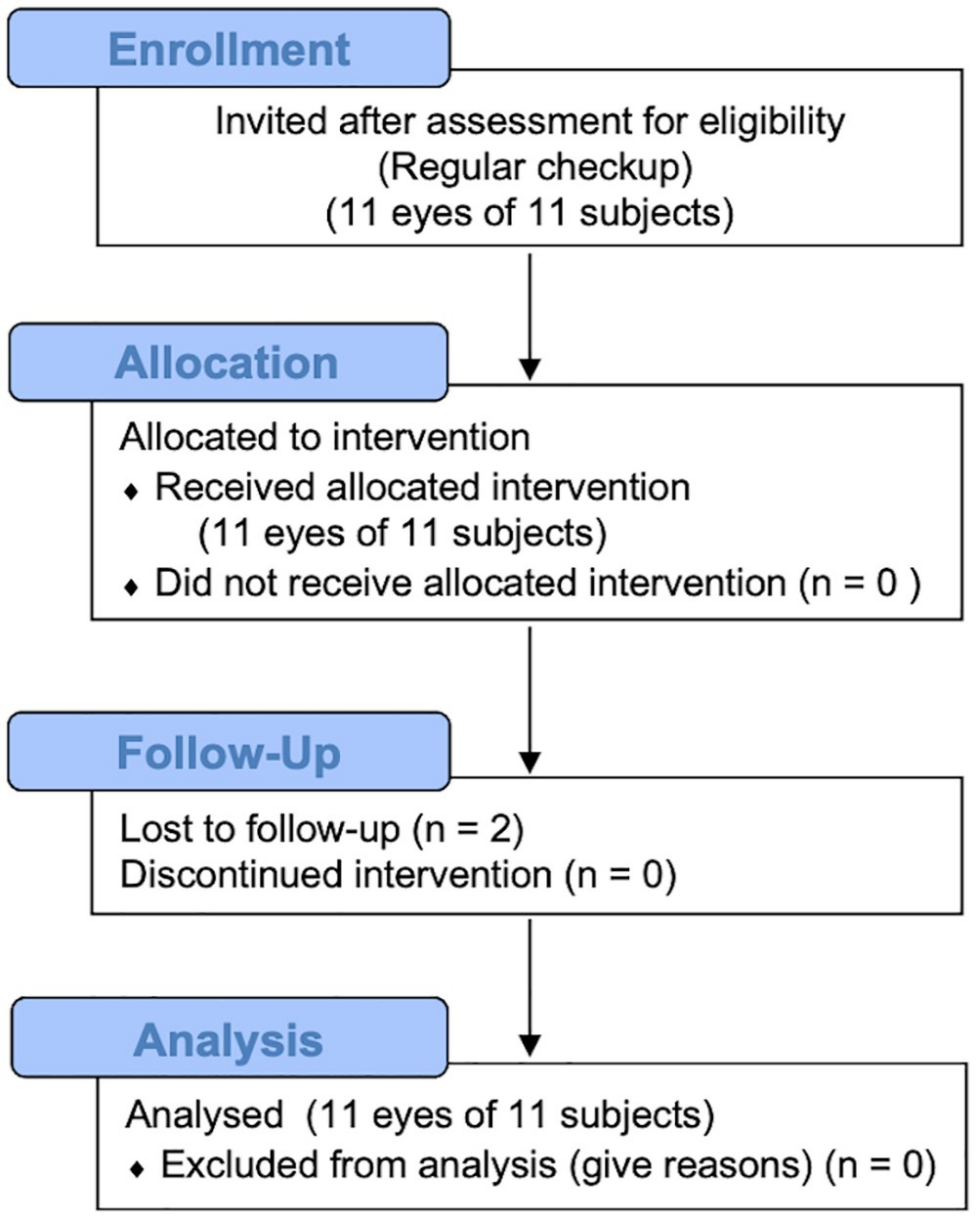
CONSORT 2010 flow diagram. (DME patients). This study included 11 eyes of 11 subjects.

In this prospective, interventional study, patients with clinically significant DME were enrolled at the Department of Ophthalmology, the University of Tokyo, Tokyo, Japan. All investigations were performed using a protocol that adheres to the tenets of the Declaration of Helsinki, and the study design was approved by the Institutional Review Boards of the University of Tokyo (approval number #11822). The consent form was obtained in writing. We also recruited healthy volunteers as controls for comparison with patients with DME. After explaining the study design, associated investigations for scientific purposes, and adjuvant imaging procedures in detail, informed consent was obtained from all subjects. This clinical study was registered (UMIN-CTR, ID: UMIN000042420, http://www.umin.ac.jp/ctr/index.htm) retrospectively due to the authors’ initial view of this non-invasive, observatory study with exploratory nature as not a clinical trial. The authors confirm that all ongoing and related trials for this intervention were registered. A total of 11 eyes each of 11 consecutive DME patients (9 men and 2 women) and 11 healthy Japanese volunteers without any history of ocular or systemic disease were enrolled (Fig 1).

In this interventional pilot study, the following inclusion criteria were employed: diagnosis of DME on the basis of the findings of fundus ophthalmoscopy, OCT, and fluorescein angiography and recurrence of DME despite anti-VEGF therapy, macular laser, sub-tenon injection of triamcinolone acetonide, or vitreous surgery. We performed SMPL as the initial treatment for these three patients as they did not consent to anti-VEGF therapy due to systemic history of stroke, medical cost, and risk of infection. Every patient underwent SMPL at the University of Tokyo Hospital between the period of December 2018 and July 2019 and was followed-up for 6 months after treatment. If required, retreatment was performed at 3 months post-treatment. The major exclusion criteria were as follows: administration of any intravitreal treatment with anti-VEGF or steroids, macular laser treatment, vitreoretinal surgery, or cataract surgery within a period of 6 months prior to baseline; application of targeted photocoagulation to retinal non-perfusion areas observed using fluorescein angiography or direct photocoagulation for the closure of microaneurysms; and significant intraocular opacity that precluded fundus examination and good-quality fundus imaging.

### Functional and morphological examinations

All subjects underwent complete ophthalmic examinations including best-corrected visual acuity (BCVA [log MAR scale]) assessment, intraocular pressure measurement, slit-lamp biomicroscopy, indirect ophthalmoscopy, color fundus photography (TRC 50IA, Topcon, Tokyo, Japan), and FAF imaging (Spectralis HRA2, Heidelberg Engineering, Heidelberg, Germany). Morphologic analysis was performed on the basis of SD-OCT (Spectralis, Heidelberg Engineering, Heidelberg, Germany) and PS-OCT scans.

### SD-OCT evaluation

**Fig 2:**
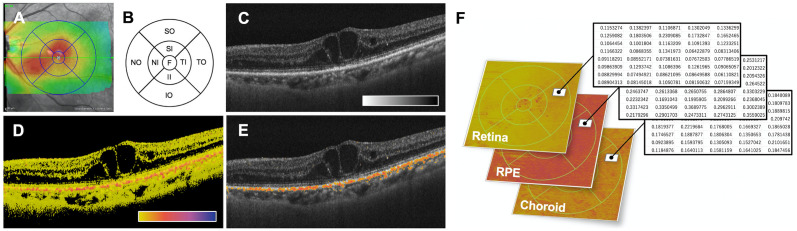
Representative SD-OCT (A), ETDRS grid (B), and PS-OCT images (C-F). (A) *En face* macular map with the ETDRS grid obtained using SD-OCT. (B) The ETDRS grid was used to demarcate nine zones delimited by solid circles with diameters 1 mm, 3 mm, and 6 mm meter centered on the fovea. (C) Intensity B-scan image [color scale: = 45 to 80 dB]. (D) Entropy B-scan image [color scale: entropy of 0 to 1]. Pixels below the detection threshold of the OCT intensity are displayed in black. (E) Overlapped with signalized entropy B-scan image (set as > 0.1) on intensity OCT image. (F) *En face* entropy map of each segmented layer (512 × 512 pixels, each) and the numeral tables, which present entropy values at each pixel location, are shown. Abbreviations in (B) are shown in the following order; TI, temporal inner; II, inferior inner; NI, nasal inner; SI, superior inner; TO, temporal outer; NO, nasal outer; SO, superior outer.

A linear 180° scan with a length of 6 mm (called “B-scans”) was performed by automatic real-time tracking with 100 frames/acquisition and an *en face* 30° × 25° macular map was generated using 31 sections. We evaluated not only the central macular thickness (CMT) but also the *en face* macular map with the ETDRS grid to assess the average retinal thickness at the sector with the greatest edema, which was the site where SMPL was mainly performed. The ETDRS grid was used to demarcate nine zones delimited by solid circles with diameters of 1 mm, 3 mm, and 6 mm, centered on the fovea; radial lines were projected onto the fundus to divide the OCT map image into nine sectors (Fig 2A and 2B). The follow-up modality was set to facilitate a perfect comparison between the linear scans and *en face* maps acquired during the observation periods.

### PS-OCT procedures and quantitative evaluation: B-scan and *En face* imaging

The detailed principles of our PS-OCT technology and the associated formula used in this study have previously been reported [27, 28]. In brief, the light source used in an optical interferometer of the PS-OCT was a frequency-swept laser (Axsun Technologies, Billerica, MA, USA); its central wavelength, sweep repetition frequency, and wavelength range were 1.05 μm, 100 kHz, and 100 nm respectively. To enable the measurement of all elements of the Jones matrix, which can characterize polarization property of the tissue completely, we used parallel-detection PS-OCT (PD-PS-OCT) for the interferometer [30]. We calculated the noise-bias-corrected polarimetric entropy of local Jones matrices as a measure of the spatial randomness of the polarization property by Cloude-Pottier decomposition [27]. Entropy of the Jones matrix is dimensionless and ranges from 0 (completely uniform) to 1 (completely random polarization). Recently, we showed that the entropy was in proportion to melanin concentration in double logarithmic scale [30].

Using the PS-OCT, the retina of each subject was scanned in a range of 6 × 6 mm with a raster scan pattern of 512 A-scans × 512 B-scans. From the Jones matrix measured by PS-OCT, we obtained an OCT intensity of the backscattered light (Fig 2C), local retardation (a phase shift between two birefringent axes), and the entropy as described above (Fig 2D). Depolarizing pixels that had entropy values > entropy_threshold_ = 0.1 were overlapped on the OCT intensity images (Fig 2E). The OCT intensity image was automatically segmented to obtain ILM layer, RPE layer, and chorioscleral interface (CSI). We then created *en face* maps of the entropy with the following definitions; retinal entropy map (averaged from ILM to 3 pixels or 13 μm above the RPE layer), RPE entropy map (averaged in 6 pixels or 26 μm centered at the RPE layer), and choroidal entropy map (averaged from 3 pixels or 13 μm below the RPE layer to the CSI). Notably, our system also provided *en face* entropy maps of these layers (512 × 512 pixels) with the ETDRS grid circle (Fig 2F). As indicated in the numerical tables in Fig 2F, we analyzed the quantitative raw data of the entropy in each section of the ETDRS grid circle.

### Treatment protocol

Macular gird laser was performed using a micropulse laser (IQ 577; Iridex Corp, Mountain View, CA, USA) with an Area Centralis^®^ contact lens (Volk Optical Inc., Menter, OH, USA). All eyes were treated using the following parameters: a spot diameter of 100 μm, 5% duty cycle of 0.2 seconds, and laser power was set to 50% of the threshold value determined in test burns. A pattern scan, with a rectangular grid consisting of 7 × 7 spots, was performed without spacing over the area of increased retinal thickness. The number of treatment spots varied according to the extent of macular edema. All procedures were performed by the same experienced clinician (K.S.).

### Outcomes and image analysis

**Fig 3:**
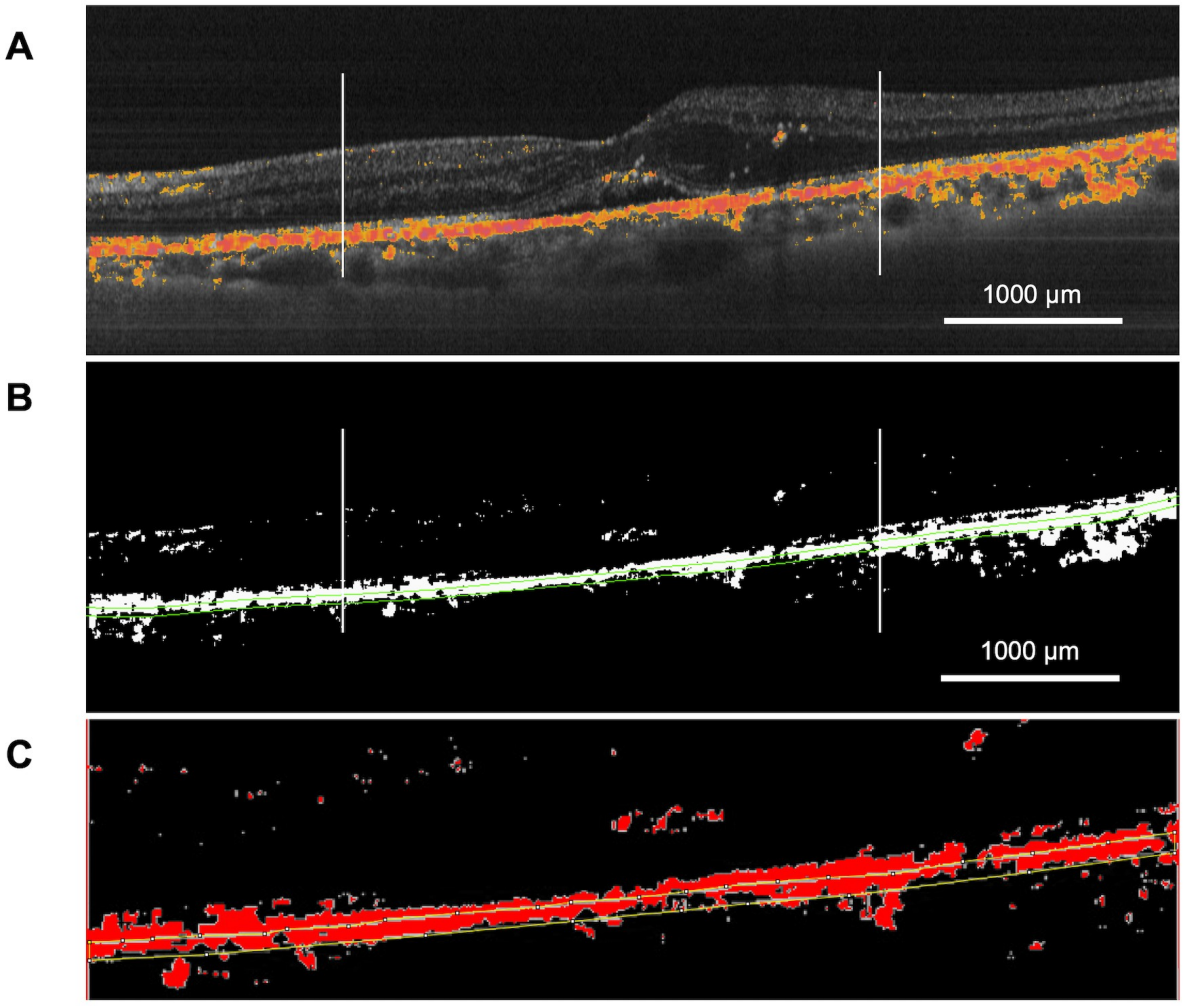
Assessment of entropy signals per unit area of RPE (pixel^2^). The linear scan image obtained by scanning through the fovea within a 1500 μm radius centered on the fovea was selected (delimited with two vertical markers traced at established distances; upper) and its imaging was binarized using Image J software (middle). Unit area of RPE was defined as 3 pixels above/below the line from automatic RPE segmentation (yellow lines), and the percentage of RPE entropy signals (red color) was calculated (bottom).

**Fig 4:**
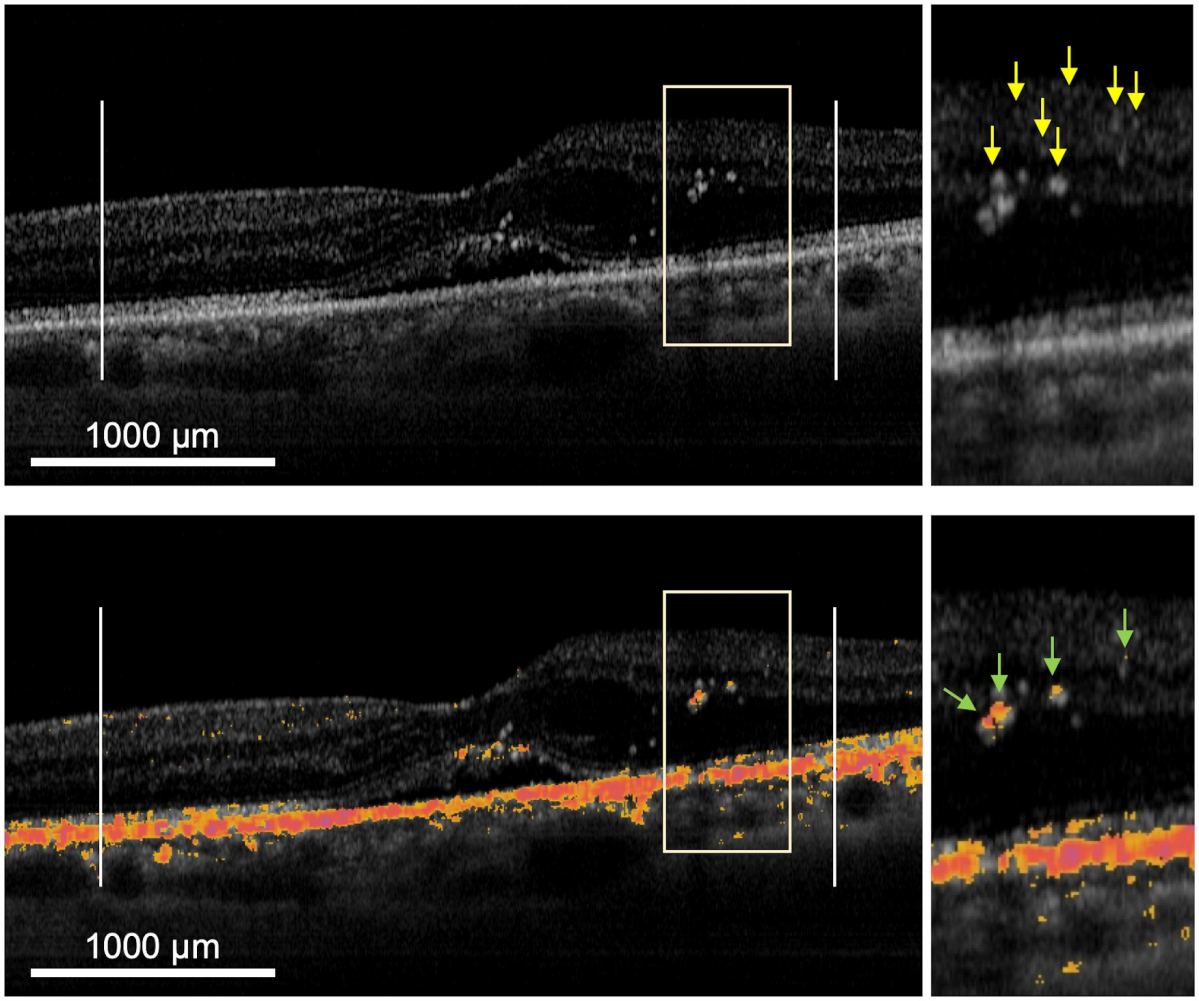
Evaluation of the number of entropy signals along with HRF. Intensity images on PS-OCT B-scan (upper) and overlapped image with signalized entropy (set as > 0.1) on its intensity OCT image (lower) of DME patient are shown. Each image obtained by scanning through the fovea within a 1500μm radius centered on the fovea was selected (delimited with two vertical markers traced at established distances, left side of each). The numbers of HRF and dots of entropy signals in the retina were evaluated. The pictures on the right side of each picture are a partial enlarged image and yellow and green arrows indicate HRS and entropy signals, respectively. HRF, hyperreflective foci; DME, diabetic macular edema.

Patients were evaluated before treatment (baseline) and at 1, 2, 3, and 6 months after SMPL. First, the changes in RPE entropy signals were calculated on the basis of PS-OCT B-scan images to evaluate the organizational characteristics of the RPE. The percentage of entropy signals per unit of RPE (pixel^2^) within a 1500 μm radius, centered on the fovea, on the fovea-spanning horizontal linear scan that was analyzed using Image J software (http://imagej.nih.gov/ij/; provided in the public domain by the National Institutes of Health, version 1.52; Bethesda, MD, U2A; Fig 3). As secondary analysis, the number of HRF and dots of high entropy thresholded at entropy threshold = 0.1 within the (sensory) retina were investigated on B-scan images. These numbers were manually counted within a 1500μm radius centered on the fovea by two blinded retina specialists (T.S. and F.A.), displayed at 400% magnification on a monitor. Subsequently, the relationship between the number of entropy signals and that of HRF was evaluated (Fig 4). Moreover, we investigated the following parameters: entropy changes after SMPL in each stratified layer including the retina, RPE, and choroid based on the ETDRS grid circle: not only the whole ETDRS grid area but also the sector with the most severe edema that was irradiated by SMPL; and the relationship between edema reduction and entropy changes.

### Statistical analysis

The results are expressed as the mean ± standard deviation. Student’s t-test was used to compare the following items between healthy subjects and patients with DME: age, BCVA, refractive error, axial length, and entropy. Mann-Whitney test was used to compare CMT and total macular volume (TMV). The Chi-square test was used to analyze sex. The Friedman test was used to analyze the changes in CMT and TMV. Moreover, area percentages of entropy per unit area of RPE (pixel^2^), the number of hyper-reflective foci in the neural retina, the percentage of entropy signals per unit area, and the changes in entropy in each retinal layer within the ETDRS grid were compared by linear mixed model analysis followed by post hoc analysis with Bonferroni adjustment for multiple comparison. Pearson linear correlation coefficient was also used to investigate correlations between edema reduction rate and entropy change rate. P values < 0.05 were considered significant: all statistical analyses were performed using R version 4.0.0 [31].

## Results

Of the total 11 DME eyes, nine patients were followed-up at 1,2,3, and 6 months after SMPL, while the remaining two patients were followed-up to 3 months after treatment. Table 1 shows the basic characteristics of patients with DME and healthy subjects, and Table 2 shows the detailed ophthalmic diagnosis and SMPL irradiation parameters. No visible signs of SMPL treatment were detected by any of the fundus imaging modalities including color fundus photography, FAF, and SD-OCT B-scan at any follow-up examination. Table 3 shows the mean values of CMT, TMV, and BCVA after SMPL. The mean CMT tended to decrease at 2 months and 6 months after treatment (P = 0.087, P = 0.18); however, TMV and BCVA did not change during the follow-up period.

**Table 1:** Basic clinical characteristics of DME patients and healthy subjects.

	Healthy subjects	DME patients	P value
No. of eyes	11	11	
Age, years	69.4 ± 3.9	73.2 ± 7.5	0.15
Sex, Men / Women	6/5	9/2	0.17
Best corrected visual acuity (logMAR)	-0.10±0	0.29±0.21	< 0.001
Refractive error (diopter), mean ± SD	-0.5±1.8	-1.0±2.3	0.64
Axial length (mm), mean ± SD	24.00±0.93	23.90±1.20	0.84
CMT, μm	221.1±19.2	407.3±105.9	< 0.001
TMV, mm^3^	8.27±0.31	9.68±1.27	< 0.01
Glycated hemoglobin, %	-	7.0 ± 0.9	
Diabetes Treatment			
Oral hypoglycemic agent, n	-	10	
Insulin, n	-	1	
Presence of medical history			
Hypertension, n	-	3	
Dyslipidemia, n	-	1	
Renal disease, n	-	0	
eGFR (ml / min)	-	66.9±17.0 (n = 7)	

Values are presented as means ± standard deviations (SD). SD-OCT (Spectralis, Heidelberg Engineering, Heidelberg, Germany) was used to examine CMT and TMV. n, number; CMT, central macular thickness; TMV, total macular volume; DME, diabetic macular edema.

**Table 2:** Details of DME patients undergoing subthreshold micropulse laser: Diagnosis and laser parameters.

No	Age, y	Sex	Diagnosis of patients (baseline)							Parameters of SMPL treatment		
			Stage of DR	Type of macular edema	CMT (μm) / TMV (mm^3^)	Previous treatment	History of photocoagulation	Lens status	BCVA (logMAR)	Laser energy (mW)	Treatment spots	Laser irradiated sector (Assigned to ETDRS grid)
1	83	M	moderate NPDR	Diffuse+SRD	358 / 11.10	None	None	Phakic	0.22	650	637	TO, TI
2	70	M	severe NPDR	Diffuse	459 / 10.05	Anti-VEGF, MAPC	PRP	Phakic	0.30	350	294	TO, TI
3	79	M	moderate NPDR	Diffuse+SRD	306 / 9.67	MAPC	focal	Phakic	0.05	400	294	SI, TI
4	66	M	severe NPDR	CME	282 / 10.50	MAPC	PRP	Phakic	0.40	400	147	TI
5	65	M	moderate NPDR	Diffuse+SRD	281 / 9.78	None	None	Phakic	0.10	400	245	TI
6	69	W	severe NPDR	CME	561 / 8.85	Vitrectomy, STTA	PRP	IOL	0.40	500	637	NI, II
7	83	W	moderate NPDR	CME	574 / 6.26	Vitrectomy, STTA	PRP	Phakic	0.30	450	294	SI, NI, II, TI
8	81	M	moderate NPDR	CME	421 / 9.28	STTA	None	IOL	0.52	550	490	NO, SI, NI
9	62	M	severe NPDR	Diffuse+SRD	290 / 9.53	MAPC	PRP	Phakic	0.15	550	441	TO, SI, NI, II
10	75	M	moderate NPDR	CME	435 / 10.82	None	None	Phakic	0.05	500	490	SO, TO, SI, II, TI
11	72	M	severe NPDR	CME	503 / 10.63	MAPC	focal	Phakic	0.70	600	588	SO, TO, SI, NI, II, TI

SD-OCT (Spectralis, Heidelberg Engineering, Heidelberg, Germany) was used to examine CMT and TMV. M, man; W, woman; NPDR, non proliferative diabetic retinopathy; SRD, serous retinal detachment; CME, cystoid macular edema; CMT, central macular thickness; TMV, total macular volume; PRP, pan retinal photocoagulation; MAPC, direct photocoagulation for microaneurysm; VEGF, vascular endothelial growth factor; STTA, sub-Tenon injection of triamcinolone acetonide; IOL, intraocular lens; BCVA, best-corrected visual acuity; TI, temporal inner; II, inferior inner; NI, nasal inner; SI, superior inner; TO, temporal outer; NO, nasal outer; SO, superior outer.

**Table 3:** Changes in SD-OCT parameters and visual acuity after subthreshold micropulse laser (n = 11).

	Baseline	1 Month	2 Month	3 Month	6 Month (n = 9)
CMT(μm)	407.3±105.9	365.1±115.2	346.6±101.3	371.3±100.4	322.1±104.3
TMV (mm^3^)	9.68±1.27	9.93±0.69	9.86±0.71	9.80±0.81	9.65±0.71
BCVA (logMAR)	0.29±0.21	0.33±0.24	0.33±0.22	0.31±0.15	0.19±0.12

Values are presented as means ± standard deviations. SD-OCT (Spectralis, Heidelberg Engineering, Heidelberg, Germany) was used to examine CMT and TMV. CMT, central macular thickness; TMV, total macular volume; BCVA, best-corrected visual acuity.

### Comparison of the mean entropy value of each *en face* layer according to ETDRS grid between healthy subjects and DME patients

**Fig 5:**
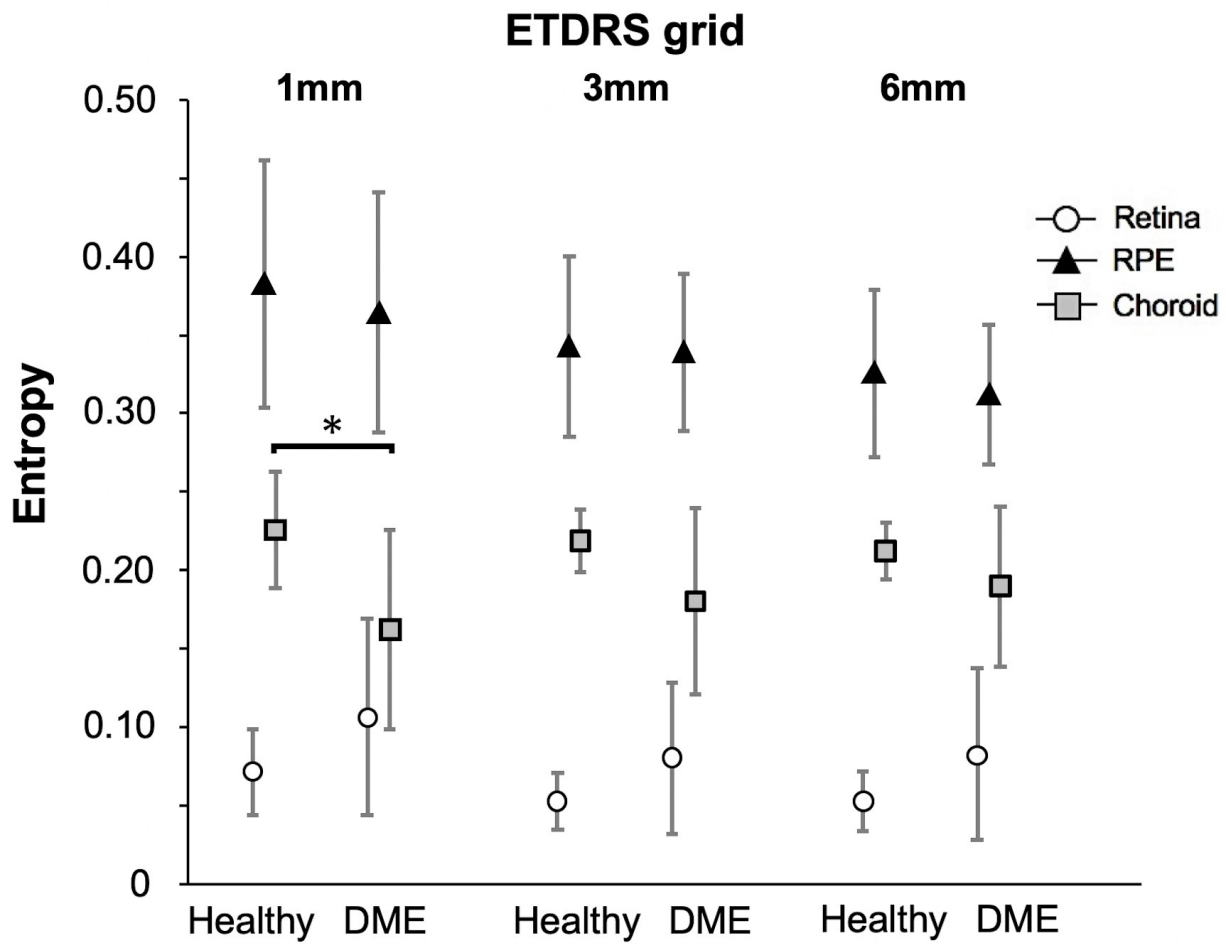
Comparison of the mean entropy value of each *en face* segmentation layers according to ETDRS grid between healthy subjects (n = 11) and patients with DME (n = 11). Mean entropy value in the choroid layer within the ETDRS circle with the diameter of 1 mm was a significantly lower in patients with DME than in healthy subjects (0.16 ± 0.064 vs 0.23 ± 0.037, P = 0.010), whereas in other circle ranges and strata, no difference was observed between patients with DME and healthy subjects. The ETDRS grid was used to demarcate nine zones delimited by solid circles with diameters of 1 mm, 3 mm, and 6 mm centered on the fovea; radial lines were projected onto the fundus to divide the OCT map into nine sectors. The results are the means ± standard deviation.

The mean entropy value in the choroid layer within the ETDRS circle of 1 mm diameter was significantly lower in patients with DME than that in healthy subjects (0.16 ± 0.064 vs 0.23 ± 0.037, P = 0.010), whereas those in other layers and circles of other diameters were not different between the two groups (Fig 5).

### Determination of changes in RPE entropy values caused by SMPL using PS-OCT B-scans

No visible signs of SMPL treatment were detected on PS-OCT B-scans images. Area percentages of entropy per unit area of RPE (pixel^2^) were 35.8 ± 17.0, 37.3 ± 22.9, 30.9 ± 18.4, 26.1 ± 9.8, and 28.2 ± 18.3 at the baseline and 1-month, 2-month, 3-month, and 6-month timepoints, respectively. Although the changes were not significant, the entropy in the RPE tended to decrease at the 3-month and 6-month time points (P = 0.14, P = 0.14).

### Determination of changes in the number of HRF and entropy signals after SMPL using PS-OCT B-scans

First, we compared the number of HRF and thresholded entropy between patients with DME and healthy subjects. The number of HRF in patients with DME was significantly higher than that in healthy subjects (74.4 ± 13.7 vs 34.0 ± 8.8, P < 0.001). In contrast, the number of thresholded entropy, HRF and thresholded entropy overlaid with HRF did not change significantly in each group (P = 0.40; 0.82, respectively).

**Fig 6:**
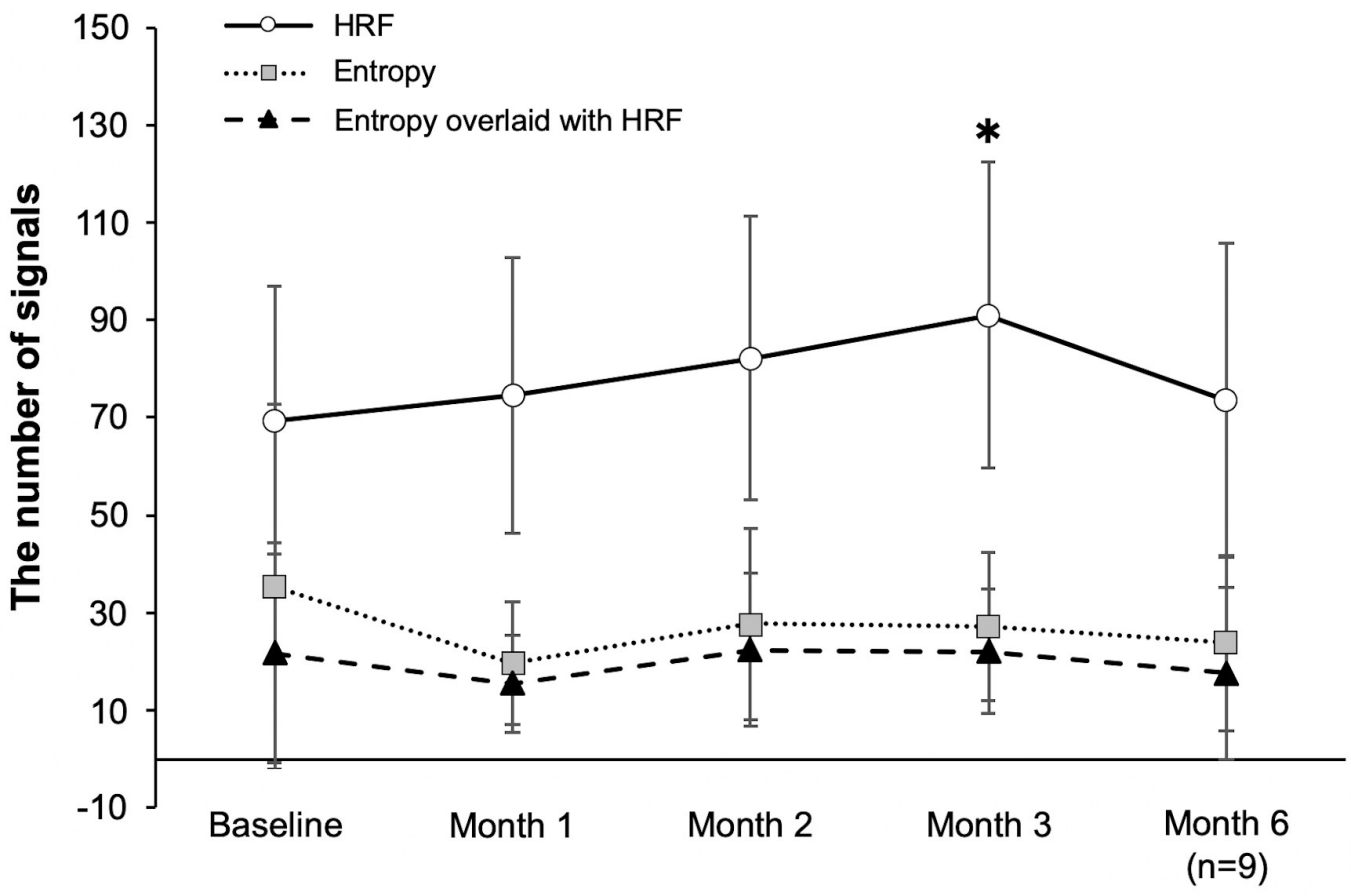
Changes in the number of HRF and entropy signals after SMPL as determined using PS-OCT B-scans (n = 11). After treatment with SMPL, there was a significant increase the number of HRF at 3-month timepoint relative to that at baseline (P = 0.019), whereas the number of entropy signals, HRF, and entropy signals overlaid with HRF did not change significantly during the follow-up. SMPL, subthreshold micropulse laser; HRF, hyperreflective foci. The results are the means ± standard deviation.

After treatment with SMPL, there was a significant increase in the number of HRF at the 3-month timepoint relative to that at the baseline (P = 0.019), whereas the number of entropy signals HRF and entropy signals overlaid with HRF did not change significantly during the follow-up period (Fig 6). In addition, no correlation was found at each endpoint between: 1) the number of entropy signals and that of HRF and 2) the number of HRF and that of entropy signals overlaid with HRF (P > 0.05, respectively). However, there was a strong positive correlation between the number of entropy signals and that of entropy signals overlaid with HRF that is, a signal with an entropy often overlaid with HRF (r = 0.98, P < 0.001 at baseline; r = 0.97, P < 0.001 at 1 month; r = 0.98, P < 0.001 at 2 months; r = 0.96, P < 0.001 at 3 months; r = 0.87, P = 0.002 at 6 months [n = 9]).

### Changes in mean entropy values of each *en face* layer according to the ETDRS grid after SMPL treatment

**Fig 7:**
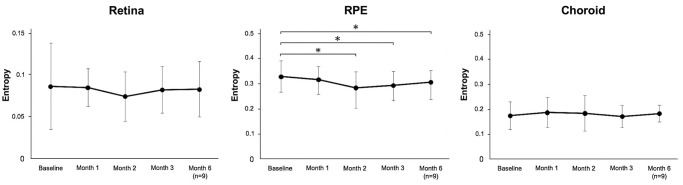
Changes in mean entropy values on SMPL-irradiated sectors according to ETDRS grid in each *en face* layer (n = 11). The mean entropy of the RPE layer tended to be lower at the 1-month timepoint (P = 0.24) and was significantly lower at the 2-month, 3-month, and 6-month timepoint after SMPL than at baseline value (P < 0.01, each). The results are the means ± standard deviation.

Among the mean entropy value changes determined based on *en face* images, that of RPE within an ETDRS grid circle of 6 mm diameter (whole area) tended to decrease from the baseline to the 2-months timepoint (P = 0.17); however, it did not reach statistical significance. There were no significant differences in entropy values in the other layers or ETDRS sectors during the follow-up. In contrast, with regard to the entropy value in SMPL-irradiated sector within the ETDRS grid, the mean entropy values in the SMPL-irradiated sector within the ETDRS grid and the mean entropy value of the RPE layer tended to decrease at the 1-month timepoint (P = 0.24), and the values were significantly lower at the 2-months, 3-months, and 6-months after SMPL than at the baseline value (P < 0.01, each) (Fig 7).

### Relationship between the rate of change of retinal thickness and that of entropy value in SMPL-irradiated sectors

**Fig 8:**
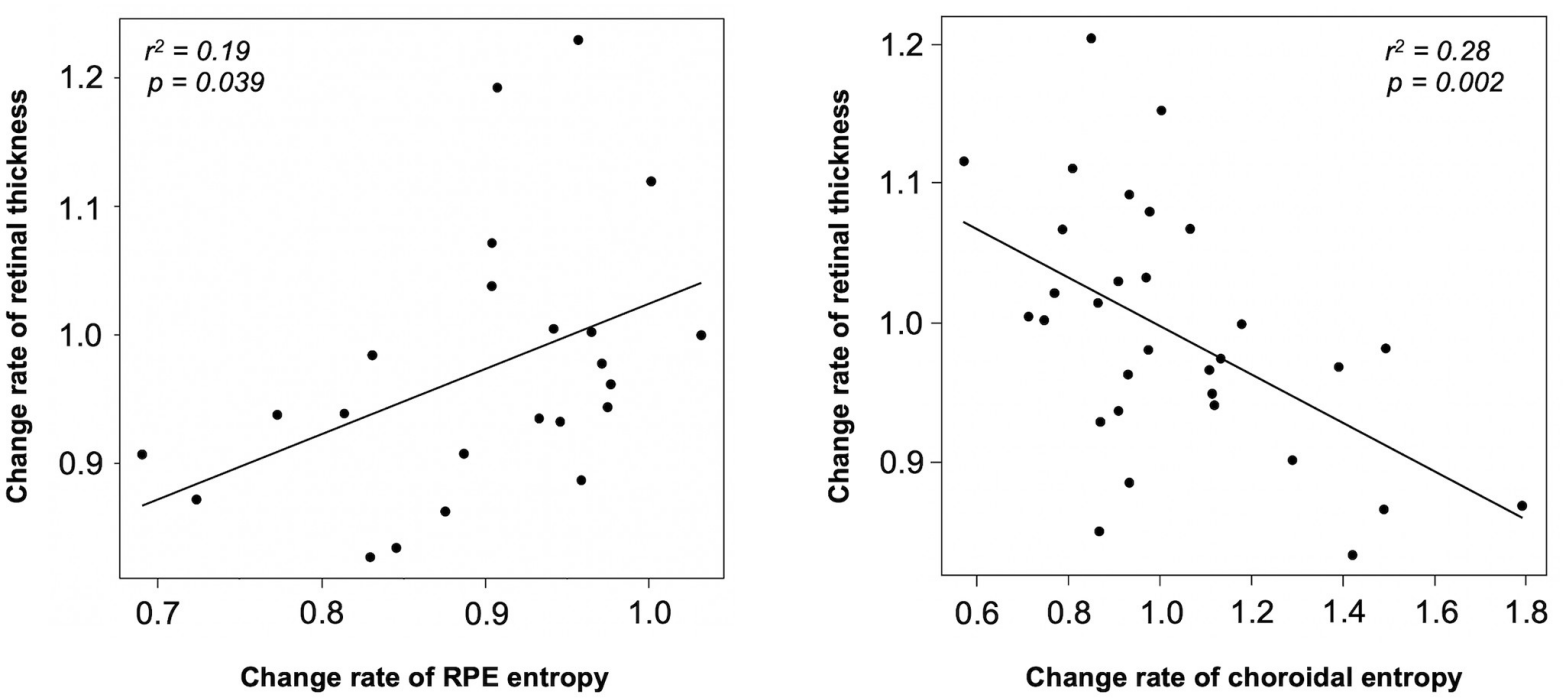
Relationship between the rate of change of retinal thickness and that of entropy value in SMPL-irradiated sectors. There was a positive correlation between the change rate of retinal thickness and that of entropy in the RPE at 6 months after SMPL (n = 9; r^2^ = 0.19, P = 0.039). On the contrary, there was a negative correlation between the change the rate of retinal thickness and that of entropy in the choroid at 3 months after SMPL (n = 11; r^2^ = 0.28, P = 0.002).

There was a positive correlation between the rate of change of retinal thickness and that of entropy in the RPE at 6 months after SMPL (r^2^ = 0.19, P = 0.039). On the contrary, there was a negative correlation between the rate of change of retinal thickness and that of entropy in the choroid at 3 months after SMPL (r^2^ = 0.28, P = 0.002) (Fig 8).

## Discussion

In this study, PS-OCT was used to investigate postoperative changes in the entropy value of the retina, RPE, and choroid, along with the effect of SMPL for DME. The results based on B-scan showed that the entropy value in RPE tended to decrease at 3 and 6 months after treatment. In addition, the entropy value did not change in each layer based on *en face* mapping within the whole ETDRS grid; however, regarding the sector where SMPL was performed, the entropy value of the RPE exhibited a significant decrease at 2, 3, and 6 months after treatment. Moreover, there was a positive correlation between the rate of changes of retinal thickness and that of entropy in the RPE within the irradiated sector at 6 months after treatment.

Unfortunately, in our study, no statistically significant changes in CMT, TMV, and BCVA were observed during the follow-up period. However, CMT tended to decrease at 2 and 6 months after treatment; this decrease is probably a result of the low relative macular thickness (five cases with CMT < 400μm) of the treated eyes, small sample size, and high standard deviation. Further studies must be conducted to confirm this result.

Previous studies have estimated, using various fundus imaging techniques including color snap images, FAF, and SD-OCT, that SMPL demonstrates no laser lesions in any of the treated areas [8, 9], which were similar to our patients. As evident from these findings, it has been difficult to elucidate the mechanism underlying the treatment success of SMPL using the conventional imaging devices. However, changes in entropy values detected using PS-OCT in the current study are potentially useful for the evaluation of the direct RPE responses and therapeutic effects by SMPL. Entropy estimated per unit area of the RPE based on B-scan images tended to decrease although no visible signs of treatment lesion were detected. In addition, although there were no changes in the mean entropy value by SMPL in the whole ETDRS grid, a significant entropy reduction was observed within the SMPL-irradiated sectors (Fig 7). Based on these findings, we hypothesized that the activation of phagocytosis by macrophages could be one possible explanation for the reduced entropy in these areas. Macrophages play a key role in innate immunity and respond rapidly to signals generated from inflamed sites. Treatment with SMPL effectively leads to the expression of HSP70 in the RPE [10], and HSP70 mediates the effects of thermal stimulation on macrophage function [32]. In diabetic retinopathy, the physiological functions of the RPE are deregulated or impaired by chronic inflammation [33]. However, the polarizing and activation of macrophages has been shown by laser irradiation [13, 34]. The present result suggests SMPL-induced thermal effects activate macrophages, which could promote phagocytosis. Consequently, impaired RPE cells are disposed; leading to a decrease in its melanin-rich RPE cells and hence, a decrease in entropy, which is detected by PS-OCT. Another possibility is thermomechanical microbubble-induced RPE cell damage as a result of SMPL irradiation. Laser-induced retinal/RPE damage is caused by thermal denaturation when the pulse durations longer than milliseconds and by microbubble formation around the melanosomes when the pulse duration is shorter than a microsecond [35]. Since the pulse duration of SMPL at 5% duty cycle is 100 μs, which does not cause irreversible heat damage to the RPE, thermomechanical microbubble-induced RPE cell damage mechanisms may be involved [35]. Melanin is eliminated due to RPE cell damage, which is associated with the reduction of entropy values at the SMPL-irradiated site, as detected by PS-OCT.

Regarding the relationship between decreased the entropy value of the RPE and reduction of macular edema in SMPL-irradiated sectors (Fig 8), we suggest the possibility of promotion of functional remodeling of RPE cells. Although melanin is lost due to moderate RPE cell damage caused by SMPL which results in entropy reduction, it is considered to contribute to cell remodeling. A previous report identified a single damaged RPE cell in the treatment area immediately after the subthreshold laser, however there were no visible signs of damage to the RPE layer in the subsequent process; that is, it is considered that RPE cells are restored under SMPL irradiation conditions [36]. For this reason, these remodeled RPE cells may contribute to DME improvement by promotion of cytokine or angiogenic factor secretion, metabolic activation, or barrier function. Another possibility could be the suppression of macrophage pro-inflammatory cytokine production. Thermal stimulation has inhibitory effects on cytokine expression in macrophages, and its effects are correlated with HSP70 or heat shock factor-1 (HSF-1) activation [37]. Previous studies using macrophage cell lines or human monocyte-derived macrophages have shown that thermal stimulation suppresses the expression of activated macrophage pro-inflammatory cytokines including TNF-α, IL-6, and IL-1β [38, 39]. These heat-induced suppressions may also involve high mobility group box1 (HMGB1), which is a nuclear protein released by activated macrophages or damaged cells and plays a role in initiating intracellular signaling and activating NF-κB and pro-inflammatory cytokine production [40]. The thermal effect has been shown to inhibit HMGB1 secretion from macrophages and increase HSF-1 and HSP70 expression. These increased levels of HSF-1 and HSP70 may reduce HMGB1 secretion and subsequent NF-κB activation and cytokine production [37]. These findings can be attributed to the fact that temporary RPE damage induced by SMPL or phagocytosis of damaged RPE by activated macrophages resulted in the loss of melanin with a reduction in entropy. However, DME was improved by the reduction of the secretion of inflammatory cytokines from macrophages. One of the therapeutic effects of SMPL include the decrease of retinal thickness by reducing the intraocular concentration of vascular endothelial growth factor [13]. We hypothesized that the reason for the low r^2^-value shown in our result may be due to such other factors aside from the changes in RPE entropy.

Previously, the characteristics of HRF scattered throughout all retinal layers, as shown by SD-OCT B-scan imaging [16, 17], and the presence of higher number of HRS in patients with diabetes than in normal subjects was reported; these findings are in line with the findings of the current study [41]. Although the origin of HRF remains unclear, previous studies have hypothesized that the deposition of extravasated lipoproteins (the precursor of hard exudates), lipid-laden macrophages, photoreceptor degeneration, activated/over-phagocytosed RPE cells, or RPE metaplasia may be involved [42–44]. In contrast, several other studies have stated that HRF is associated with inflammatory responses in the retina [41, 45], with representation aggregates of activated macrophages [46]. In our study, changes in the number of entropy signals as well as HRF were determined after SMPL based on PS-OCT B-scan imaging. The number of HRF increased significantly 3 months after SMPL but decreased thereafter (Fig 6). Hard exudates tend to consolidate and eventually clear by laser treatment [47]. We considered that HRF increased up to 3 months after SMPL due to aggregation of activated macrophages or excessively phagocytosed RPE cells, or other immune responses; however, as these effects peaked, HRF began to decrease with the absorption of hard exudates. In general, since laser-induced inflammation occurs at an earlier stage, our results did not support the fact that HRF is associated with inflammatory response; but some other immune responses may be involved instead. In contrast, the number of entropy signals remained unchanged after SMPL, and no correlation was found between the number of entropy signals and that of HRF. Therefore, the number of entropy signals, as determined using PS-OCT B-scan, could not be shown as a therapeutic effect of SMPL. Since its association with HRF remains unclear, further studies are needed. In contrast, HRF and entropy signals were observed at the same sites. As entropy is also sensitive to melanin, HRF may also represent melanin itself or melanin-phagocytosed macrophages.

The mean entropy value of the choroid layer within the ETDRS circle with a diameter of 1 mm was significantly lower in patients with DME than in healthy subjects (Fig 5). Various studies have described the choroidal structure using swept source-OCT; however, the changes in choroidal thickness associated with diabetic retinopathy remain controversial. Previous studies have shown that the choroid thickness of patients with DME tends to increase [48] or remain unchanged [49]; while others exhibit decreased choroidal thickness [50]. As the characteristics of the choroid in patients with diabetes remain poorly understood, further studies are needed for the interpretation of entropy-related results obtained in the current study.

The study was limited by its small sample size, thus a type II error (false negative) might occur. In addition, the short follow-up period and the possibility that entropy was affected by confounding individual systemic or ocular local factors were also cited as limitations of this study. Furthermore, it is conceivable that studies with experimental models are needed to obtain a more precise understanding of entropy signals. Nevertheless, our study suggests that entropy measured using PS-OCT may be a new parameter for objective monitoring of SMPL-induced functional RPE changes that could not previously be assessed directly.

## Supporting information

S1 Appendix For research participants. (DOCX)

S2 Appendix Research ethics review application. (DOCX)

S3 Appendix TREND statement checklist. (DOCX)
