# Supplementary material for: Changes in entropy on polarized-sensitive optical coherence tomography images after therapeutic subthreshold micropulse laser for diabetic macular edema: A pilot study
Source: PLoS One. 2021 Sep 13;16(9):e0257000. doi: 10.1371/journal.pone.0257000 (PMC8437304; doi:10.1371/journal.pone.0257000)
Supplement: S1 Appendix — (DOCX) [file pone.0257000.s001.docx]

**※Please note that this document was provided to the study subjects in Japanese. It is translated into English for the purpose of this manuscript.**

**For Research Participants**

For participating in the "Exploratory Research Using Polarized-Sensitive OCT”

1. Overview of this study

**[Research Topic]**

Exploratory study using polarized-sensitive OCT

**[Research institute and principal investigators]**

The institutions and principal investigators for this research are listed below:

- Research Organization: Department of Sensory and Functional Medicine, Department of Surgery, Graduate School of Medicine, The University of Tokyo
- Principal Investigators: Satoshi Kato, Associate Professor, Department of Ophthalmology
- Responsibilities: General supervision of the research

**[Collaborative research institutions]**

TOMEY Corporation (lead agency)

Satoshi Sugiyama, Manager

**[Study period]**

From the time of the Ethics Review Committee's approval, until March 31, 2021

**[Research Objective]**

This study aims to investigate the structure of the eye and the mechanisms of eye disease using a new instrument called polarized-sensitive OCT. Polarized sensitive OCT is a modification of the OCT machine currently used in clinical practice to examine the structure of the eye. Existing OCT machines examine the structure of the eye by irradiating a faint laser beam to the back of the eye and detecting the reflected light, but depending on the condition of the tissue at the back of the eye, it may not detect the structure accurately. This problem was solved by devising a method for detecting the reflected light with polarized-sensitive OCT, which is more accurate than the existing OCT systems. It has also been shown that the improved accuracy of OCT has the potential to reveal the location of specific tissues in the eye, such as melanin and fibers, which could not be examined before. The use of this polarized-sensitive OCT in the examination of various eye diseases is expected to lead to new discoveries that may lead to better treatments. The safety of using the polarized-sensitive OCT is thought to be the same as that of existing OCTs, which are currently proven to be very safe.

**[Research Method]**

Patients who are considered to benefit from understanding the structure of the ocular fundus by OCT in the outpatient ophthalmology clinic of the University of Tokyo Hospital and who have various diseases such as glaucoma, high myopia, various macular diseases, retinal degenerative diseases, uveitis, ocular tumors, etc., or healthy volunteers will be included in this study.

In this study, the eyes will be imaged with polarized-sensitive OCT to produce various images. By looking at these images together with other examinations performed in the usual practice, we will examine whether new knowledge can be obtained that will lead to a clarification of the structure of the eye and the mechanisms of diseases that have not been previously understood. Since a large amount of data will be generated, we will ask for the cooperation of TOMEY Corporation, the developer of the machine, for analysis. In this study, we will use the information in the medical record, such as images, examination data, and basic diseases and medical history, which are obtained in the usual medical practice. If there is any change in the medical condition during the course of the examination, polarized-sensitive OCT imaging is performed after consultation with the patient.

In the case of conducting imaging on healthy volunteers, polarized-sensitive OCT imaging is performed after confirming that there is no obvious abnormality in the eye through several regular examinations. This will allow us to compare the findings with those of the diseased eye and to examine findings that may lead to early detection of the disease and clarification of its pathogenesis.

Research participants will be allowed to view the research protocol and materials related to the research methods as long as it does not interfere with protecting the personal information of other research participants or ensuring the originality of the research.

1. Voluntary research cooperation and freedom of withdrawal

It is up to you, the participant, to decide whether or not you wish to participate in this study.

If you wish to withdraw your consent, you must sign the withdrawal form and submit it to the physician in charge of the outpatient department or to Takahiro Minami, the contact person for this study. Please note that failure to cooperate with the study will not be detrimental to you. At your request (or that of a family member if you are a minor), we will destroy the samples, information, data, etc. and the results of the study if possible. However, please note that we cannot discard if the results of the research have already been published in a paper or in other forms of publication.

1. Protection of personal information

All specimens, information and data collected in this research must be handled with care to prevent them from being disclosed to the outside world.

Your samples and data will be sent to TOMEY Corporation for analysis and storage, however, your name, address, and date of birth will be deleted and replaced with a new code to make it impossible to identify you. The code is kept strictly in a password-locked personal computer for the use of administrative personnel only. However, if necessary, our laboratory can restore the sign to the original name, etc.

1. Publication of research results

We will publish the results of our research in conference presentations and in academic journals and databases, after ensuring that your name and other personal information is not revealed. If we receive a personal inquiry, we will disclose your personal results or the overall results (or both).

1. Benefits and disadvantages to research participants

It is not likely that this study will provide you with any immediately useful information. However, the results of this study are expected to contribute to the future development of research in multiple areas of ophthalmology. Therefore, it is likely to benefit you in the sense that it may present you with better treatment options in the future.

The disadvantage is the time commitment of a few minutes required for additional testing. The safety of the machine is comparable to existing OCTs and is considered to be very safe. The degree of invasion is minor and we do not anticipate any health hazards; however, in the unlikely event of disability, the insurance will cover the cost of the procedure.

1. Policy for handling materials (specimens) after the completion of research

The human specimens, information and data you provide will be used only for the purpose of this study. However, if you agree, we will continue to store them as a valuable resource for future research, even after completion of the study. The samples, information, data, etc. will be securely stored in a password-locked personal computer that can only be used by the person in charge of managing personal information, with a sign so that the identity of the samples, information, data, etc. cannot be determined.

In the future, if the specimens, information and data are to be used for any new research or provided to other research institutions, we will obtain approval from the Ethics Committee of the University of Tokyo School of Medicine again.

1. Expenses

We will not ask you to pay for the costs of this research, but you will be responsible for your own expenses in your normal practice. There will be no honorarium.

1. Ownership of intellectual property rights arising from research

Although there is a possibility that patents and other rights may arise as a result of this research, these rights belong to the state, research institutes, joint research organizations including private companies, and research workers, and you have no rights to these patents and any other rights. In addition, you shall have no rights to the economic benefits that may arise from the patents, etc.

1. Others

This research was approved by the Ethics Committee of the University of Tokyo's School of Medicine and conducted with the permission of the University of Tokyo's Graduate School of Medicine and Dean of the School of Medicine. Expenses for this research have been paid for by TOMEY Corporation.

In this research, we received research funds from the Japan Agency for Medical Research and Development (AMED) for the theme "Development and Commercialization of a Next-Generation Polarized Light-Sensitive OCT for Understanding Ocular Diseases from Multiple Perspectives," as well as one polarized-sensitive OCT machine and an analysis program from TOMEY Corporation. We have reported to the Conflict of Interest Advisory Committee of the University of Tokyo's School of Medicine to properly manage any conflicts of interest.

We will receive a polarized-sensitive OCT from TOMEY Corporation to conduct this study, but we will not intentionally lead them to make the study or report the results in their favor.

If you have any comments or questions, please feel free to send them to the following:

**[Contacts]**

Principal Investigators:

Satoshi Sugiyama (TOMEY Corporation)

Satoshi Kato (Department of Ophthalmology, The University of Tokyo)

Contact person:

Takahiro Minami (Department of Ophthalmology, The University of Tokyo)

7-3-1 Hongo, Bunkyo-ku, Tokyo 113-0033, Japan

Department of Ophthalmology, Department of Sensory and Motor Medicine, Department of Surgery, Graduate School of Medicine, The University of Tokyo

Tel: 03-5800-8660, ext. 33497, Fax: 03-3817-0798

**Consent form**

To the Dean of the Graduate School of Medicine, The University of Tokyo,

Research theme: "Exploratory research using polarized OCT" (Examination No. 11822)

At the time when I participated in the above research, I received an explanation about the items described in the explanatory document and fully understood this, so I agree to become a research participant in this research.

Yes No

I received explanations and understood the following items:

□ About the outline of this research

□ Voluntary research cooperation and freedom of withdrawal

□ About protection of personal information

□ Publication of research results

□ Benefits and disadvantages to research participants

□ Handling policy for materials (samples) after the research is completed

□ About your cost burden

□ Others

In addition, the materials (samples) related to me will be used for new research planned and implemented in the future.

I agree to save the period and use it for research:

Yes No

(Saved after the end of this research) (Discarded at the end of this research)

Date: yyyy/mm/dd

Name (research participant or substitute) (self-signed) ＿＿＿＿＿＿＿＿＿＿＿＿＿＿

(In the case of a substitute, the relationship with the person) ＿＿＿＿＿＿＿＿＿＿＿＿＿
