# Supplementary material for: Changes in entropy on polarized-sensitive optical coherence tomography images after therapeutic subthreshold micropulse laser for diabetic macular edema: A pilot study
Source: PLoS One. 2021 Sep 13;16(9):e0257000. doi: 10.1371/journal.pone.0257000 (PMC8437304; doi:10.1371/journal.pone.0257000)
Supplement: S2 Appendix — (DOCX) [file pone.0257000.s002.docx]

**※Please note that this document was provided to the study subjects in Japanese. It is translated into English for the purpose of this manuscript.**

Form 1-1 　　　　 11822-(2)

Research Ethics Review Application

Last updated: ver. 31, October 9, 2019

Date received: October 9, 2019

To: Dean of the Graduate School of Medicine and Faculty of Medicine at the University of Tokyo

Name of applicant (research director): Satoshi Kato

Affiliation/title: Associate Professor, Department of Ophthalmology and Vision Correction

Phone (extension) number: 30462

E-mail: [katou-s@ka2-so-net.ne.jp](mailto:katou-s@ka2-so-net.ne.jp)

I hereby submit the application for the ethics review for the following research.

Research title Exploratory Research Using Polarized OCT

Keyword Intervention study

Name, affiliation, title, etc. of the research staffs:

| (Name) | (Affiliation) | (Title) | (Extension) | (Research ethics seminar completion No. and expiry period) |
| --- | --- | --- | --- | --- |
| Toshikatsu Kaburaki | Department of Ophthalmology and Vision Correction | Associate Professor | 37499 | 2018-3399 (03/31/2020) |
| Megumi Honjo | Department of Corneal Transplantation | Assistant Professor | 30463 | 2018-4228 (03/31/2020) |
| Ryo Obata | Department of Ophthalmology and Vision Correction | Assistant Professor | 36823 | 2018-4440 (03/31/2020) |
| Makoto Aihara | Department of Ophthalmology and Vision Correction | Professor | 30460 | 2018-4349 (03/31/2020) |
| Takahiro Minami | Department of Ophthalmology and Vision Correction | Student at the Graduate School | 33497 | 2018-5225 (03/31/2020) |
| Koji Ueda | Department of Ophthalmology and Vision Correction | Post doctoral Fellow | 30464 | 2018-4065  (03/31/2020) |

Classroom manager/Head of clinical department:

Name: Makoto Aihara

Affiliation: Department of Ophthalmology and Vision Correction

Person in charge of contact:

Name: Satoshi Kato

Affiliation: Department of Ophthalmology and Vision Correction

Phone number: (Extension) 30462

E-mail: katou-s@ka2.so-net.ne. jp

Research Protocol

1. Research title

Evaluation of the usefulness and challenges in the clinical use of polarized-sensitive OCT for fundus

1. Research overview
   1. Background and objective

Diseases of the retina/choroid and surrounding tissues, the mechanism and predisposition for their onset and progression in vivo have not been fully elucidated, nor has there been a treatment method that guarantees a sufficiently good visual prognosis. Under these circumstances, more detailed observation of the fundus is needed to further elucidate the pathogenesis of the disease and to detect it at an early stage, and the development race to solve this problem is underway in Japan and abroad.

Since 2000, optical coherence tomography (OCT) has gradually spread to the general practice, making it possible to obtain cross-sectional images of the eye and greatly improving the quality of ophthalmic clinical practice. The energy of the laser used in OCT is sufficiently low to provide a safe and reliable method of examining the eye. However, it is thought that the polarization state of the reflected light depends on the local conditions of the reflected light in the tissue, and existing OCT cannot accurately detect the light intensity depending on the polarization state of the reflected light. For example, when the anterior segment of the eye is imaged by OCT, the image sometimes appears as if the Schlemm's canal were in the vicinity of the Schlemm's canal, which has a structure called the Schlemm's canal, but this has been shown to be an artifact. In addition, when imaging the choroid, artifacts that resemble non-existent choroidal structures often appear.

In the present study, we have developed a new OCT (hereinafter referred to as "polarized-sensitive OCT") that can accurately detect the light intensity regardless of the polarization state of the reflected light by using the same light source as the existing OCT and improving the detection system of the reflected light. Polarized-sensitive OCT is expected to avoid the artifacts described above and provide more accurate images. It has also been shown that two types of tissues, melanin and fibers, each have their own characteristic polarization state, and it is possible to extract and visualize the components of melanin and fibers from the information in the reflected light. In ophthalmology, there are important melanin-related diseases such as macular degeneration and Harada's disease, and fiber-related diseases such as glaucoma, and the use of polarized-sensitive OCT is expected to provide new insights into the pathogenesis of various ophthalmic diseases including these. The use of polarized-sensitive OCT is expected to provide new insights into the pathogenesis of various ophthalmic diseases, including these diseases. The purpose of this study is to use polarized-sensitive OCT exploratory in clinical settings and to observe cross-sectional images of the ocular fundus to further understand the pathology of the disease and to evaluate the usefulness of polarized-sensitive OCT and its current issues.

- 1. Methods

Patients who have visited the outpatient ophthalmology clinic of the University of Tokyo Hospital and for whom it would be useful to understand the pathophysiology of the condition of the patient by OCT will be recruited by the physician in charge during the outpatient clinic. Specifically, the clinical research will be explained in a written explanation, and patients who have given their consent and signed a consent form will be eligible for this clinical study. In the case of minors or adults who are unable to give informed consent in person, the consent signature of a guardian or guardianship or other equivalent will be required. The fundus will be imaged with polarized-sensitive OCT in the subject's case as well as with normal OCT.

Various images are created from the data obtained by polarized-sensitive OCT. These include specific images obtained from the polarization information. By comparing the accumulated data and images, as appropriate, with information obtained from existing anatomical and pathological knowledge and other examinations (including conventional OCT), we will examine whether information unique to polarized-sensitive OCT can be obtained and whether the image preparation algorithm is valid. In particular, it may be possible to quantify the artifacts in the existing OCT images when comparing polarized OCT with existing OCT. As for the statistical methods to be used in the analysis, since various hypotheses may be made in the process of collecting data in an exploratory manner, we will conduct a rational statistical analysis accordingly.

Study period and eligible period: 04/01/2008 to 03/31/2021
Research period: after approved to 03/31/2021

- 1. Subjects and documents (samples), etc.

1. Subjects

[Selection criteria]

Patients

Patients with outpatients for whom outpatient observation of the fundus using polarized-sensitive OCT would be useful in understanding the pathophysiology of the disease, specifically, glaucoma, intense myopia, various macular diseases, retinal degenerative diseases, uveitis, ocular tumors, and other diseases, who consent to participate in this clinical study will be included in this study. There is no specific age limit, but in the case of minors or adults who are unable to give their informed consent, a signature of a guardian or guardianship or other equivalent will be required.

[Exclusion criteria] There are no exclusion criteria for this project.

[Subjects who require ethical consideration] None

[Number of subjects] 2500 cases

Healthy person (Volunteer)

Men and women over 20 years of age, about 15 for each of the 10 years of age, up to the age of 90

[Number of subjects] 100 cases

Documents (samples), etc.

Polarized-sensitive OCT data of the fundus, image data created by analyzing this, images obtained in normal medical care, examination data, basic information such as gender and date of birth, and general clinical information described in medical records such as medical history

- 1. Actual experiences of the research participants (subjects, research subjects)

[Actual experiences]

(1) At the time of the outpatient consultation, each subject eligible for the study will receive a written explanation of the details of the study from the physician in charge of the outpatient clinic, sign the consent form, and receive a copy of the written explanation. However, if the patient is a minor or an adult who is unable to give informed consent in person, his or her guardian or someone equivalent to the guardian will sign the consent form.

(2) The patient is moved to the polarized-sensitive OCT station located in the outpatient clinic, sits in a chair and places his/her face on the chin strap of the polarized-sensitive OCT and follows the examiner's instructions to observe a fixed viewpoint inside the polarized-sensitive OCT.

The examiner supports the upper eyelid with his fingers to prevent the upper eyelid from resting on the pupil in cases of ptosis.

(4) Hold still for a few seconds.

(5) The examination is completed when the patient's face is removed from the chinrest, and at the same time, the actual experience of the clinical study is over.

The experience in (2) to (5) is the same as for normal OCT imaging.

[The number of times the examination is performed] One examination per outpatient session.

[The number of examinations] One examination is performed per outpatient visit. If necessary during follow-up, the examination should be performed on a case-by-case basis.

[Invasion] As mentioned above, the test should not exceed a minor invasion, as in the case of conventional OCT.

[Discontinuation criteria] If the patient expresses the intention to withdraw, or if it is considered necessary to discontinue the study owing to problems.

1. Research facilities and their roles
2. Names of applicable facilities within and outside the school and their roles (may be attached separately)
3. Facility for the obtainment of informed consent: Outpatient or Inpatient Building at Department of Ophthalmology and Vision Correction, the University of Tokyo Hospital
4. Facility for the collection or ownership of personal information and documents, etc.: Medical Office at Department of Ophthalmology and Vision Correction, the University of Tokyo Hospital
5. Facility for the anonymization of documents, etc.: Inside the main body of outpatient polarized-sensitive OCT at the University of Tokyo Hospital
6. Facility for the analysis of documents, etc.: Medical Office and Laboratory No. 5 at Department of Ophthalmology and Vision Correction, the University of Tokyo Hospital, TOMEY corporatrion
7. Facility for the storage of documents, etc.: Medical Office and Laboratory No. 5 at Department of Ophthalmology and Vision Correction, the University of Tokyo Hospital
8. Handling in external facilities and the status (may be attached separately)

Not applicable

1. Ethical considerations in the research
   1. Informed consent
2. Method

- Method for providing explanation to the research participants: Using the written information (attached document)
- Method for obtaining consent from research participants, or clearly expressing the intention for “non-participation in the research”: Using the consent form and consent withdrawal document

1. Need for consideration in research participations who require ethical consideration, and measures to implement:

■Yes (fill in details)□No

For yes, circle the applicable item number and indicate the method of handling.

1. Minors 2. Adults without sufficient decision-making capability

Implementation details

In an outpatient setting, patients for whom observation using polarized-sensitive OCT would be meaningful in understanding the condition are given a verbal explanation using an explanatory document. Afterwards, the patient is given the explanatory document and asked to sign a consent form and receive a copy of the form at the same time. However, if the patient is a minor or an adult who is unable to give informed consent, the consent should be signed by the patient's guardian or someone equivalent to the guardian. Patients for whom observation using polarized-sensitive OCT is useful in understanding the pathology of their condition are, specifically, patients with various diseases such as glaucoma, high myopia, various macular diseases, retinal degenerative diseases, uveitis, and ocular tumors.

- 1. Protection of personal information

Personal information

Personal information will be handled within the school.

■Yes

Personal identification information handled

□Name ■Sex □Initials ■Patient ID ■Date of birth □Telephone number

□Address □E-mail address □Other

Documents containing the personal identification information handled

■Image data (photographs) □Biological samples □Case report form

■Other (visual acuity test results)

□No

I have confirmed again with the joint developer that the documents (samples) do not include personal information.

□Yes □No

*Tick the following only if personal information is being handled.

Basic responsibilities relating to personal information, etc.

1. Protection of personal information, etc.

With regard to the handling of personal information, we will comply with the regulations in the ethical policies, as well as the conditions established in the Act on the Protection of Personal Information (Law No. 57, 2003) and the Act on the Protection of Personal Information Held by Independent Administrative Agencies, etc. (Law No. 59, 2003)

■Yes □No

1. Appropriate means of obtainment, etc.
   1. We have not obtained personal information for this research by false or other dishonest means.

■Yes □No

2-2 We do not handle personal information obtained in this study beyond the scope of prior agreement with the research subjects.

■Yes □No

Security management

1. Appropriate handling
   1. The personal information obtained for this research in the ownership of the research institution affiliated with the applicable researchers (including those cosigned for storage) will be handled appropriately to avoid leakage, loss, damage, and for other security management.

■Yes □No

- 1. The research director (name: Satoshi Kato) will appropriately manage the personal information.

■Yes □No

- 1. Method of protection personal information

All documents (samples) containing personal information will be anonymized (personal information, etc. will be deleted from the image data, and verbatim records will be prepared from voice data with anonymization of proper nouns.

■Yes

■Linkable anonymization will be performed

(1) Storage of personal information:

Duration (If the consent of the research participants is obtained, it will continue to be stored as a valuable resource for future research even after the research is completed.)

Location (Inside the main body of outpatient polarized-sensitive OCT at the University of Tokyo Hospital)

Method (Save with the password-protected computer that came with the polarized OCT main unit)

(2) Disposal of personal information:

Timing: If the consent of the research participants is obtained, it will continue to be

stored as a valuable resource for future research even after the research is completed)

Method: (It is stored in the personal computer inside the polarized OCT body at least 5 years after the end of the research period, but if it is necessary to destroy it, the storage device of the computer attached to the polarized OCT body is physically destroyed)

□Unlinkable anonymization will be performed.

(1) We understand that all personal information must be deleted at this point.

□Yes □No

□No

□Anonymization will not be performed (this includes direct transcription of original data, such as medical terminal device, to the document for sending, with the documents transcribed immediately deleted)

(1) Personal information will not be extracted from the medical terminal device and stored.

□Yes □No

- 1. Personal information will be deleted for documents sent to parties outside the school.

■Yes □No □Not applicable

1. Organization of systems and supervision, etc. for security management
   1. Organizational security management measures
2. Control of personal information will be enforced within the affiliated classroom.

■Yes □No

1. The manager of the affiliated classroom is aware of the research title under which the personal information is handled.

■Yes □No

- 1. Human security measures

1. The staff members involved in this research understands the knowledge relating to anonymization and procedures for publication.

■Yes □No

- 1. Physical security measures

1. Personal information will be stored in a personal computer within a lockable room, a file service within the hospital medical terminal device, or locked in a locker, to prevent theft.

■Yes □No

- 1. Technical security measures

1. The electronic files containing personal information will be locked with a password and stored in a standalone personal computer locked with password, or in a file service within the hospital medical terminal device.

■Yes □No

1. Measures to prevent malware are implemented for personal computers used for the handling of personal information.

■Yes □No

- 1. Handling of documents, etc. within the school that does no involve personal information
- Storage location of documents (samples), etc. and the name of person responsible for storage: Satoshi Kato
- Timing and method of disposal

Data after linkable anonymization will be stored by Satoshi Kato, the research director, in a standalone personal computer not connected to LAN, located in the Laboratory of Department of Ophthalmology and Vision Correction, locked with a password. Store it strictly for 5 years or more on a password-locked personal computer that can only be used by the person in charge of personal information management. We do not anticipate disposal at this time, but we will dispose of the storage device in an appropriate manner (assuming erasure or destruction) when it becomes necessary.

- Possibility of use outside this research title: None
- Possibility of use by parties other than the staff involved in this research: None

1. Safety assurance
2. Hazards and discomforts that may affect the research participants through this research

It is not expected that the research participants will be at risk from conducting this research. Discomfort can result from those time constraints, as it can take minutes (actual shots are seconds) and waiting time to perform additional tests, which is a normal OCT. Similar to inspection, usually no problem. Countermeasures can be explained carefully so that they can be understood.

1. Measures to take for risks and discomfort, etc.

No particular health risk to study participants is envisioned. In addition, short-term restraint due to research participation is a burden on the patient. On the other hand, it may be in the interest of society that new findings can be obtained by carrying out this research, leading to the elucidation of pathological conditions and the development of useful medical devices. We consider that this study is ethically acceptable when we weigh these things.

1. Disclosure of research results to research participants

- Disclosure to research participants: Yes; if a personal inquiry is received, the personal results or the overall results (or both) will be disclosed.
- Method of disclosure: Scheduled presentation at an academic conference and submission of a research paper.

1. Remarks, etc.

Trust accounts of the Department of Ophthalmology will be used as research funds.

The research format is joint research between the Department of Ophthalmology at the University of Tokyo and one corporation; the name of the company is “QD Laser, Inc.” No financial payment is provided to the participants in this research. Also, there is no conflict of interest for disclosure with regard to this research.

Research Fund Sources and Conflicts of Interest

1. Funds from public institutions

Japan Agency for Medical Research and Development (AMED) The issue adopted in the 2017 Medical-Engineering Collaboration Commercialization Promotion Project "Next to grasp eye diseases from various angles" The subsidy received in "Development and Commercialization of Generation Polarization Sensitive OCT" (a consignment contract is signed between TOMEY Corporation, which is the representative organization of the above business, and the University, and the funds are transferred to the University in the form of consignment costs. This is all covered by AMED subsidies)

2) Provision other than funds

□ None

■ Free provision [Name of pharmaceuticals / medical devices Polarized OCT 1 unit]

■ Labor provision [Details Tome Corporation creates a program. ] □ Other []

3) Situation regarding conflicts of interest

□ There is no conflict of interest to disclose

■ Disclosure
